# Association Between Social Media Use and Cancer Screening Awareness and Behavior for People Without a Cancer Diagnosis: Matched Cohort Study

**DOI:** 10.2196/26395

**Published:** 2021-08-27

**Authors:** Lei Qin, Xiaomei Zhang, Anlin Wu, James S Miser, Yen-Lin Liu, Jason C Hsu, Ben-Chang Shia, Linglong Ye

**Affiliations:** 1 School of Statistics, University of International Business and Economics Beijing China; 2 Guanghua School of Management, Peking University Beijing China; 3 College of Medical Science and Technology, Taipei Medical University Taipei Taiwan; 4 Taipei Cancer Center, Taipei Medical University Taipei Taiwan; 5 Department of Pediatrics, City of Hope National Medical Center Duarte, CA United States; 6 Department of Pediatrics, Taipei Medical University Hospital Taipei Taiwan; 7 Department of Pediatrics, School of Medicine, College of Medicine, Taipei Medical University Taipei Taiwan; 8 International Ph.D. Program in Biotech and Healthcare Management, College of Management, Taipei Medical University Taipei Taiwan; 9 Graduate Institute of Business Administration, College of Management, Fu Jen Catholic University New Taipei City Taiwan; 10 Artificial Intelligence Development Center, Fu Jen Catholic University New Taipei City Taiwan; 11 School of Public Affairs Xiamen University Xiamen China

**Keywords:** social media, cancer screening awareness, cancer screening behavior, gender-specific effects, propensity-score matching, general population

## Abstract

**Background:**

The use of social media in communications regarding cancer prevention is rapidly growing. However, less is known about the general population’s social media use related to cancer screening awareness and behavior for different cancers.

**Objective:**

We aimed to examine the relationship between social media use and cancer screening awareness and behavior among people without a cancer diagnosis.

**Methods:**

Data were collected from the Health Information National Trends Survey 5 Cycle 1 to 3 in the United States (n=12,227). Our study included 10,124 participants without a cancer diagnosis and 3 measures of screening awareness (those who had heard of hepatitis C virus [HCV], human papillomavirus [HPV], and the HPV vaccine) and 4 measures of behavior (those who had prostate-specific antigen tests, Papanicolaou tests for cervical cancer, as well as breast cancer and colon cancer tests). Propensity-score matching was conducted to adjust for the sociodemographic variables between the social media user and nonuser participants. Multivariable logistic regression was used to assess the association of social media use by gender. Jackknife replicate weights were incorporated into the analyses.

**Results:**

Of the 3794 matched participants, 1861 (57.6% weighted) were male, and the mean age was 55.5 (SD 0.42) years. Compared to social media nonusers, users were more likely to have heard of HCV (adjusted odds ratio [aOR]=2.27, 95% CI, 1.29-3.98 and aOR=2.86, 95% CI, 1.51-5.40, for male and female users, respectively) and HPV (aOR=1.82, 95% CI, 1.29-2.58 and aOR=2.35, 95% CI, 1.65-3.33, for male and female users, respectively). In addition, female users were more likely to have heard of the HPV vaccine (aOR=2.06, 95% CI, 1.41-3.00). No significant associations were found between social media use and prostate-specific antigen tests in males, Papanicolaou tests and breast cancer tests in females, or colon cancer tests in both male and female users.

**Conclusions:**

While social media services can potentially promote cancer screening awareness in the general population, but they did not improve screening behavior after adjusting for socioeconomic status. These findings strengthened our understanding of social media use in targeting health communications for different cancers.

## Introduction

The introduction of screening for early prostate, colorectal, breast, and cervical cancer detection has significantly reduced mortality rates over the past few decades [1]. However, cancer remains the second leading cause of death in the United States [2], and premature cancer deaths resulted in US $94.4 billion lost earnings among people aged 16 to 84 years in 2015 [3]. Therefore, there is growing concern regarding the low proportion of cancer screening awareness and behavior in the general population and the relative disparities in cancer screening awareness and behavior due to race and ethnicity and socioeconomic status.

A recent National Health Interview Survey reported that the utilization of recommended cancer screenings is far lower than the Healthy People 2020 targets for the nation [4]. However, cancer screening awareness and behavior disparities exist according to cancer types, race and ethnicity, socioeconomic status, and other health care access factors [1,4,5]. For example, from 2005 to 2015, colorectal cancer screenings have increased steadily, but prostate, breast, and cervical cancer screenings have declined [6]. In addition, prostate, cervical, breast, and colon cancer screening rates are lower among Hispanic and Asian groups than non-Hispanic White and Black groups, declining with decreasing education levels [6]. Therefore, new channels for informing target populations are needed to improve the cancer screening awareness and behavior regarding different cancers and reduce the relative disparities.

The use of social media has increased over the past decade and has become a new channel for promoting cancer prevention [7]. Compared to older approaches to cancer interventions with unidirectional and paternalistic outreach barriers, contemporary social media is easy to access, bidirectional, interactive, and patient-driven [7,8]. Social media services are widely used, varied, and continually innovating, offering substantial opportunities for health communication [9]. Given these inherent advantages of social media, there has been rapid growth in the use of different social media services by health care providers and the general population to communicate information regarding cancer prevention, such as basic cancer knowledge, healthy lifestyles, and the importance of cancer screenings.

Previous research on the use of social media in cancer prevention has primarily focused on educational material [7]. In most previous studies, general information regarding cancer screening has been reported as a key component of cancer prevention education. However, a single study has addressed the detailed descriptions of bowel preparation for colorectal cancer screening [7,10]. Among these studies, cancer screening awareness and behavior for different cancers were not thoroughly examined [7]. Thus, more insight is needed to understand better the effects of social media on peoples’ awareness and behavior concerning the screening for different cancers.

Despite the diversity of social media services, the previous studies mainly used specific types of social media, including Facebook, Twitter, and WeChat (a widely used social media app in China) [10-12]. Although one qualitative study reported that Facebook use helped promote breast cancer screening awareness [12], inconsistent effects of different social media services were found for the screening behavior for different cancer types [10,11]. Rosemary et al [11] showed that using Twitter did not improve specific behaviors regarding breast cancer prevention, including screening. In contrast, one study revealed an improvement in colorectal screening behavior through the use of WeChat [10]. However, the discussions are still too limited to provide comprehensive information to determine and compare the effects of social media on cancer screening awareness and behavior for different cancer types.

The variety of evolving social media services makes it necessary to examine how the general use of social media impacts cancer screening awareness and behavior for different cancer types. Moreover, most users of different social media services vary across racial and ethnic minority and socioeconomic groups. Only certain populations were included in previous prevention studies using specific types of social media [7,10-12]. Even less is known about the general population’s social media use related to cancer screening awareness and behavior for different types of cancer.

This study aimed to assess how the general use of social media impacted people’s cancer screening awareness and behavior for different cancer types in a nationally representative sample of US adults without a cancer diagnosis. In addition, because of the inherent heterogeneity of different cancer types between males and females, the gender-specific effects of social media use were examined. By evaluating social media use in the general population, this study furthers our understanding of the relationship between health communications using social media and health-promoting behaviors.

## Methods

### Data Sources

This study adopted data from the National Cancer Institute (NCI) Health Information National Trends Survey (HINTS), including HINTS 5 Cycle 1 (2017, n=3285), HINTS 5 Cycle 2 (2018, n=3504), and HINTS 5 Cycle 3 (2019, n=5438). The HINTS is a nationwide survey of noninstitutionalized individuals aged 18 years and older in the United States and has been conducted every 1 to 2 years by the NCI since 2003. It uses a probability-based sampling with a 2-level stratified design, considering areas with high or low concentrations of minorities. Data from the 3 surveys were collected by mail or Web Pilot from January to May 2017, 2018, and 2019, respectively. Data were available in a publicly accessible repository that does not issue DOIs. Publicly available data sets were analyzed in this study. More detailed information on the study design and high-quality HINTS data have been published elsewhere [13]. After excluding those individuals with a cancer diagnosis, 10,124 participants remained. Participants with missing data for any variables were excluded from the final analyses, resulting in a sample of 7090 social media users and 2775 social media nonusers.

### Measurements

#### Social Media Use

Social media use was assessed using all 5 subquestions of the B14 question in the HINTS questionnaire (“Sometimes people use the internet to connect with other people online through social networks like Facebook or Twitter. This is often called ‘social media.’”). The 5 subquestions asked, “In the past 12 months, have you used the internet for any of the following reasons: (1) to visit a social networking site, such as Facebook or LinkedIn; (2) to share health information on social networking sites, such as Facebook or Twitter; (3) to write in an online diary or blog (ie, weblog); (4) to participate in an online forum or support group for people with similar health or medical issues; or (5) to watch a health-related video on YouTube?” The participants answered either “yes” or “no,” and those using social media were deﬁned by a “yes” response to any of the 5 questions.

#### Cancer Screening Awareness and Behavior

The measures of cancer screening awareness consisted of 3 dependent variables, which were assessed by 3 questions: (1) “Have you ever heard of the hepatitis C virus (also known as Hep C or HCV)?”; (2) “Have you ever heard of HPV?”; (3) “Before today, have you ever heard of the cervical cancer vaccine or HPV shot?” Participants indicated their response with either a “yes” (1) or “no” (0).

Cancer screening behavior measures contained 4 dependent variables defined by 4 questions: (1) “Have you ever had a prostate-specific antigen (PSA) test?” (1=yes; 0=no); (2) “How long ago did you have your most recent Papanicolaou (Pap) test to check for cervical cancer?” (1=I have had a Pap test [a year ago or less, 1 to 2 years ago, 2 to 3 years ago, 3 to 5 years ago, or more than 5 years ago]; 0=I have never had a Pap test); (3) “When did you have your most recent mammogram to check for breast cancer, if ever?” (1=I have had a mammogram [a year ago or less, 1 to 2 years ago, 2 to 3 years ago, 3 to 5 years ago, or more than 5 years ago]; 0=I have never had a mammogram); (4) “Have you ever had one of these tests, including colonoscopy, sigmoidoscopy, and stool blood test to check for colon cancer?” (1=yes; 0=no).

Due to the differences in the survey content between the different Cycles, the data for HCV were from Cycle 2 (2018) and Cycle 3 (2019), the data for HPV and the HPV vaccine were from Cycles 1 to 3 (2017-2019), the data for PSA testing were from Cycle 1 (2017) and Cycle 3 (2019), the data for Pap and breast cancer testing were from Cycles 1 to 3 (2017-2019), and the data for colon cancer testing were from Cycle 2 (2018) and Cycle 3 (2019).

#### Sociodemographic Covariates

The sociodemographic characteristics in this study included self-reported measures of gender (male or female), age, race and ethnicity (non-Hispanic White or racial and ethnic minority), education level (high school or less or more than high school), income (less than US $20,000 or US $20,000 or more annually), and geographic area (nonmetropolitan or metropolitan). Measurements of the geographic area were obtained by 9 metropolitan codes corresponding to the Rural-Urban Continuum Codes with a range of 1 to 9 [14]. Based on the cutoff points adopted by the United States Department of Agriculture and previous HINTS studies, this study divided the geographic areas into metropolitan (codes 1 to 3) and nonmetropolitan (codes 4 to 9) areas [14-16].

### Statistical Analysis

This study compared the sociodemographic characteristics of social media users and nonusers via chi-squared tests for categorical variables and 2-tailed *t*-tests for continuous variables. Propensity-score matched analysis was carried out to adjust the sociodemographic variables between the social media users and nonusers. Confounders used for matching social media users and nonusers included gender, age, race and ethnicity, education, income, and geographic area. Given the gender differences for different cancer types, the differences in cancer screening awareness and behavior were compared by the social media use of males and females. Crude odds ratios (cOR), adjusted odds ratios (aOR), and their 95% CIs stratified by gender were computed using univariate and multivariable logistic regression analyses to clarify the impact of social media use on each dependent variable. This study adjusted the multivariable analyses for potential confounders, including gender, age, race and ethnicity, education, income, and geographic area.

All statistical analyses were carried out using SAS (version 9.4; SAS Institute). Given the complex survey design of the HINTS, jackknife replicate weights were incorporated into the analyses to obtain population-level estimates. The jackknife method creates a set of replicate samples from the original sample and provides an estimate of the variable and its variance of interest [17,18]. A *P*<.05 was considered statistically signiﬁcant.

## Results

### Sociodemographic Characteristics and Social Media Use

All percentages, means, and standard errors reported in this section are the weighted values. Among 10,124 participants without a diagnosis of cancer, 50% (n=4103) were male, and the average age was 48 (SD 0.20) years (Table 1). Nearly two-thirds (n=5551, 59%) of the participants were non-Hispanic White, and about one-third (n=2496, 31%) had a high school education or less. The income of about 18% (n=1715 ) of the participants was less than US $20,000 annually. In addition, 14% (n=1246) of the population lived in nonmetropolitan areas. Among the 9868 participants with complete information, 72% (n=7093) used social media. There were statistically significant differences in all sociodemographic characteristics between the social media user versus nonuser participants (all *P*<.001). Compared to social media nonusers, the social media users were more likely to be female (56%, n=1291), tended to be younger (mean age 44 years vs 59 years), non-Hispanic White (n=4129, 61% vs n=1344, 54%), were educated above a high school level (76%, n=5765 vs n=1580, 50%), and were more likely to live in metropolitan areas (n=6298, 88% vs n=2361, 83%).

**Table 1:** Sociodemographic characteristics for social media users and nonusers. All percentages, means, and standard errors reported in the table are the weighted values.

Characteristics		Total (N=10,124)	Social media use (n=9868)		
			Use (n=7093)	Nonuse (n=2775)	*P* value^a^
**Gender, n (%)**					
	Male	4103 (49.4)	2723 (47.3)	1291 (56.4)	<.001
	Female	5895 (50.6)	4308 (52.7)	1433 (43.6)	
Age (years), mean (SE)		47.6 (0.20)	44.1 (0.25)	58.8 (0.56)	<.001
**Race and ethnicity, n (%)**					
	Non-Hispanic White	5551 (58.6)	4129 (60.7)	1344 (53.5)	<.001
	Racial and ethnic minority	4573 (41.4)	2964 (39.3)	1431 (46.5)	
**Education, n (%)**					
	≤High school	2496 (30.7)	1277 (24.4)	1108 (49.6)	<.001
	>High school	7479 (69.3)	5765 (75.6)	1580 (50.4)	
**Income (US), n (%)**					
	<$20,000	1715 (18.0)	909 (14.9)	732 (27.6)	<.001
	≥$20,000	7397 (82.0)	5662 (85.1)	1616 (72.4)	
**Geographic area, n (%)**					
	Nonmetropolitan	1246 (13.5)	795 (12.4)	414 (17.5)	<.001
	Metropolitan	8878 (86.5)	6298 (87.6)	2361 (82.5)	

^a^Chi-squared tests for categorical variables and *t*-tests for continuous variables.

### Sociodemographics of Participants After Propensity-Score Matching

After propensity-score matching, a total of 3794 (33%) participants (1897 social media users and nonusers each) remained (Table 2). Of those 1864 (58%) were male, 2110 (59%) were non-Hispanic white, 1278 (45%) had a high school education or less, 907 (24%) had an annual income below US $20,000, and 528 (16%) lived in nonmetropolitan areas. No statistically significant differences between social media users and nonusers were found in any of the sociodemographic characteristics.

**Table 2:** Sociodemographic characteristics between social media users and nonusers matched by the propensity-score method. All percentages, means, and standard errors reported in the table are the weighted values.

Characteristics		Total (n=3794)	Social media use (n=3794)		
			Use (n=1897)	Nonuse (n=1897)	*P* value^a^
**Gender, n (%)**					
	Male	1861 (57.6)	941 (57.2)	920 (58.0)	.77
	Female	1933 (42.4)	956 (42.8)	977 (42.0)	
Age (years), mean (SE)		55.5 (0.42)	55.6 (0.49)	55.5 (0.69)	.98
**Race/ethnicity, n (%)**					
	Non-Hispanic White	2110 (59.1)	1066 (59.2)	1044 (59.0)	.93
	Racial and ethnic minority	1684 (40.9)	831 (40.8)	853 (41.0)	
**Education, n (%)**					
	≤High school	1278 (45.3)	656 (46.8)	622 (43.9)	.33
	>High school	2516 (54.7)	1241 (53.2)	1275 (56.1)	
**Income (US), n (%)**					
	<$20,000	907 (23.5)	442 (23.9)	465 (23.0)	.74
	≥$20,000	2887 (76.5)	1455 (76.1)	1432 (77.0)	
**Geographic area, n (%)**					
	Nonmetropolitan	528 (16.4)	287 (17.2)	241 (15.6)	.42
	Metropolitan	3266 (83.6)	1610 (82.8)	1656 (84.4)	

^a^Chi-squared tests for categorical variables and t-tests for continuous variables.

### Cancer Screening Awareness and Behavior by Social Media Use and Gender

Male social media users, compared to nonusers, were more likely to have heard of HCV (n=394, 92% vs n=321, 84%; *P*=.02) and HPV (n=502, 56% vs n=398, 43%; *P*=.003). However, awareness of the HPV vaccine was not significantly different between male social media users and nonusers (n=420, 48% vs n=369, 41%, respectively; *P*=.12). For all 1861 male participants, including both social media users and nonusers, about 815 (49%) had taken PSA tests (*P*=.97), and 488 (55%) of the social media users had taken colon cancer tests compared to 433 (47%) social media nonusers (*P*=.09; Table 3).

For the 1933 female participants, the social media users, compared to nonusers, were more likely to have heard of HCV (89%, n=402 vs 75%, n=336; *P*<.001), and HPV (72%, n=683 vs 56%, n=566; *P*≤.001), and HPV vaccine (72%, n=664 vs 58%, n=577; *P*≤.001). In addition, 97% (n=913) social media users and 94% (n=921) nonusers had taken Pap tests (*P*=.31), 87% (n=849) social media users and 85% (n=874) nonusers had taken breast cancer tests (*P*=.43), and 66% (n=514) social media users and 64% (n=479) nonusers had taken colon cancer tests (*P*=.61).

**Table 3:** Differences in cancer screening awareness and behavior by social media use and gender. All percentages, means, and standard errors reported in the table are the weighted values.

Characteristics		Social media use						
		Male (n=1861)				Female (n=1933)		
		Use (n=941)	Nonuse (n=920)	*P* value^a^	Use (n=956)		Nonuse (n=977)	*P* value
**Awareness**								
	Heard of HCV^b^ (yes), n (%)	394 (91.7)	321 (83.5)	.02	402 (89.3)		336 (75.0)	.002
	Heard of HPV^c^ (yes), n (%)	502 (56.4)	398 (43.4)	.003	683 (72.3)		566 (55.5)	<.001
	Heard of HPV vaccine (yes), n (%)	420 (48.0)	369 (40.5)	.12	664 (71.7)		577 (57.7)	<.001
**Behavior**								
	Had PSA^d^ test (yes), n (%)	427 (49.0)	388 (49.2)	.97	N/A		N/A	
	Had Pap^e^ test (yes), n (%)	N/A	N/A		913 (96.5)		921 (94.1)	.31
	Had breast cancer test (yes), n (%)	N/A	N/A		849 (87.1)		874 (85.0)	.43
	Had colon cancer test (yes), n (%)	488 (54.8)	443 (46.7)	.09	514 (65.7)		479 (64.0)	.61

^a^Chi-squared tests for categorical variables and t-tests for continuous variables.

^b^HCV: hepatitis C virus.

^c^HPV: human papillomavirus.

^d^PSA: prostate-specific antigen.

^e^Pap: Papanicolaou.

### Impact of Social Media Use on Awareness and Behavior of Cancer Screening

Table 4 reports the relationship between social media use and cancer screening awareness and behavior in male and female participants presented by weighted analyses. Male social media users were more likely to have heard of HCV (cOR 2.17, 95% CI 1.25-3.77; aOR 2.27, 95% CI 1.29-3.98) and HPV (cOR 1.68, 95% CI 1.20-2.36; aOR 1.82, 95% CI 1.29-2.58) compared to nonusers. No statistically significant effects of social media use on awareness of the HPV vaccine and taking PSA tests or colon cancer tests were observed in males.

**Table 4:** Univariate and multivariable logistic regression assessing the impact of social media use on cancer screening awareness and behavior. Weighted analyses are presented.

Characteristics			Male (n=1861)									Female (n=1933)						
			cOR^a^ (95% CI)		*P* value		aOR^b^ (95% CI)		*P* value		cOR (95% CI)			*P* value		aOR (95%CI)		*P* value
**Awareness**																		
	Heard of HCV^c^	2.17 (1.25-3.77)		.007		2.27 (1.29-3.98)		.005		2.80 (1.57-4.99)			<.001		2.86 (1.51-5.40)		.002	
	Heard of HPV^d^	1.68 (1.20-2.36)		.003		1.82 (1.29-2.58)		.001		2.10 (1.53-2.87)			<.001		2.35 (1.65-3.33)		<.001	
	Heard of HPV vaccine	1.35 (0.92-1.98)		.12		1.42 (0.98-2.06)		.07		1.85 (1.34-2.57)			<.001		2.06 (1.41-3.00)		<.001	
**Behavior**																		
	Had PSA^e^ test	0.99 (0.68-1.46)		.97		1.17 (0.73-1.88)		.52		N/A					N/A			
	Had Pap^f^ Test	N/A				N/A				1.72 (0.57-5.19)			.33		1.54 (0.58-4.10)		.38	
	Had breast cancer test	N/A				N/A				1.20 (0.76-1.88)			.43		1.06 (0.70-1.60)		.78	
	Had colon cancer test	1.38 (0.95-2.02)		.09		1.58 (0.98-2.54)		.06		1.08 (0.80-1.45)			.62		1.24 (0.88-1.74)		.21	

^a^cOR: crude odds ratio.

^b^aOR: adjusted odds ratio.

^c^HCV: hepatitis C virus.

^d^HPV: human papillomavirus.

^e^PSA: prostate-specific antigen.

^f^Pap: Papanicolaou.

Female social media users were more than twice as likely to have heard of HCV (cOR 2.80, 95% CI 1.57-4.99; aOR 2.86, 95% CI 1.51-5.40), HPV (cOR 2.10, 95% CI 1.53-2.87; aOR 2.35, 95% CI 1.65-3.33) or the HPV vaccine (cOR 1.85, 95% CI 1.34-2.57; aOR 2.06, 95% CI 1.41-3.00) compared to nonusers. There was no statistically significant impact of social media use on taking Pap tests, breast cancer tests, and colon cancer tests.

## Discussion

### Principal Results

Our study conducted a comprehensive evaluation of the effects of general social media use on cancer screening awareness and behavior among US adults without a cancer diagnosis. Although the impact of social media on screening awareness varied across different cancer types and genders, we showed social media could promote cancer screening awareness in the general population. However, it did not improve screening behavior after adjusting for race and ethnicity and socioeconomic status. In light of the growing use of social media by health care providers and the general population, the findings of this study can contribute to targeted messaging for cancer prevention and reduce disparities between the different groups.

After matching and controlling for sociodemographic characteristics, we found that the general social media use had a significant impact on the awareness of HPV in both male and female adults. In contrast, its effect on HPV vaccine awareness was only significant in female adults. Similar to an online cervical cancer prevention study, HPV awareness can be increased through social media [19]. Persistent infection with specific HPV subtypes accounts for almost all cervical cancers in women, practically all anal cancers (over 9 out of 10), most vaginal, oropharyngeal, vulvar, and penile cancers (between two-thirds and three-quarters), and some oral cavity and laryngeal cancers in both men and women [20]. In addition to cervical cancer, most HPV-associated cancers could be reduced by current vaccines [20]. Hence, HPV vaccination is recommended for both males and females at specific ages. The American Cancer Society suggests that all children aged 11 or 12 years should be vaccinated against HPV infections. HPV vaccination with Gardasil 9 (Merck) has been approved for men and women aged 26 to 45 years by the United States Food and Drug Administration since 2018 [1,21]. An Australian study suggested that Twitter-derived measures of information exposure were associated with HPV vaccine coverage for both adolescent males and females [22]. Hence, in addition to communicating about HPV vaccination and cervical cancer prevention, social media can provide additional health information about other HPV-associated cancers and target the male population.

A significant impact of social media use on HCV awareness was also found for both male and female adults in this study. A southeast Michigan study on health disparities in hepatitis C revealed a suboptimal impact of multiple sociodemographic factors on hepatitis C screening and care [23]. Through the internet and a personal health record training program, health-related internet use increased among low-income patients with HCV, and their self-efficacy and patient activation were also improved [24]. As a patient-driven internet service, social media emerges as a tool linking the general population with HCV awareness. However, its impact on improving hepatitis C screening and care among specific groups at high risk of HCV requires further research.

Social media use did not significantly impact cancer screening behavior, including taking PSA tests in male adults, Pap tests and breast cancer tests in female adults, or colon cancer tests in both male and female adults. In contrast, previous studies regarding specific social media services in the United States or China showed inconsistent effects of social media on cancer screening behavior [10-12]. These results may be due partly to misinformation in cancer communications commonly shared via social media.

There are increasing concerns about misleading or inaccurate medical science information on social media [25-27]. For example, a recent study on the accuracy of genitourinary malignancy articles shared on social media showed inaccurate and misleading articles were shared at a significantly higher rate on average than accurate articles, and 1 in 10 articles about PSA testing contained misinformation [28].

In addition, a lack of consensus on the clinical use of cancer screening and treatment may cause unnecessary panic when shared via social media. The so-called gray area of PSA testing with a high rate of false positives has resulted in its questionable clinical usefulness in Italy [29]. Prostate cancer tends to develop slowly, and its overdiagnosis and overtreatment are controversial [30]. Overdiagnosis and overtreatment have the potential for unnecessary side effects, including physical and psychological harms. The decrements in quality of life from side effects may offset some of the gains in length of life obtained from screening [31,32].

Given that social media amplifies scientific information and misinformation [25], a Japanese study [33] has revealed the information war for and against cancer screening messages has begun online. Therefore, accurate information with more engagement from scientists or professional institutions on social media is urgently needed to prevent misinformation about cancer screening from expanding and to achieve the screening target rates recommended in Healthy People 2030. In addition, collaboration with celebrities diagnosed with cancer may trigger substantial social media interest compared to traditional efforts for raising cancer screening awareness and affect screen behaviors, allowing health care providers to leverage social media methods like celebrities.

In terms of routine cancer care, PSA or colon cancer tests for males and Pap tests and breast cancer tests for females are part of routine check-ups. However, it may not be easy to change the behavior of people with negative perceptions regarding cancer screenings. As several HPV vaccine studies have pointed out, prior exposure to negative information was correlated with the later expression of negative opinions [34], and among Twitter users in Canada, the United Kingdom, and Australia, those with negative or opposing views about vaccinations were more likely to be better connected than those with positive views [35], leading to more barriers to improving routine cancer screening. As for cancers and tests that are not part of routine care, such as skin cancer [31], social media could be used to increase prior exposure to positive views for targeted populations to raise screening rates.

The findings in this study also suggest that among social media users, increased health information awareness did not lead to increased cancer screening behavior. The gap between “knowing” and “doing” in cancer screening behavior may be associated with the social characteristics of social media. Vaterlaus et al [36] reported a significant impact of social media on diet and exercise, which are commonly shared on social media. Compared to diet and exercise, health communication about cancer screening targets relatively small groups and has less engagement. Breast Cancer Awareness Month and associated events on Twitter revealed that a strategic communication plan calls for ongoing social media conversations on breast cancer and screening mammograms to help increase breast cancer screening rates [11].

The reach of social media is substantial, and the demographics of health-related users vary across different social media platforms [37]. It is valuable to promote cancer screening through various social media platforms according to the demographics of their health-related users to increase cancer screening rates for certain socioeconomic groups and hard-to-reach populations. Combined with ads targeted toward specific audiences, social media platforms could provide potentially innovative and effective ways to communicate positive health information.

### Limitations

This study had several limitations. First, this study was based on the general use of social media rather than health-related use, which may result in measurement errors. Given the wide use of social media, it is difficult to separate health communications from the general use of social media. For instance, COVID-19 dominated social media communications at the beginning of 2020. Furthermore, the degree of social network use (eg, frequency and duration) was not quantified, and thus, the time-dependent effects of social media use could not be measured. Second, this study defined the use of Pap tests and breast cancer tests via “yes” or “no” responses among all the participants, without considering the recommended screening frequency by age. Moreover, the patterns of social media use vary across different age groups. Further studies on the effects of social media use on the frequency of cancer screening awareness and behavior at different ages should be conducted.

### Conclusions

This study identified the association between social media use and cancer screening awareness and behavior among people without a cancer diagnosis after matching and controlling for sociodemographic factors. Social media use was associated with HPV and HCV awareness among both male and female participants, and the awareness of the HPV vaccine among female participants. No significant correlation was observed between social media use and screening behavior, including PSA, Pap, breast cancer, or colon cancer test-taking. This study provided a comprehensive assessment of the association between general social media use and cancer screening awareness and behavior for different types of cancer in the general population. Our study suggests that health communication using social media can effectively impact the awareness of specific cancers. Still, a more innovative and targeted approach is needed, including accurately delivering messages to hard-to-reach populations and improving specific screening behaviors.

## Abbreviations

aOR: adjusted odds ratios
cOR: crude odds ratios
HCV: hepatitis C virus
HINTS: Health Information National Trends Survey
HPV: human papillomavirus
NCI: National Cancer Institute
Pap: Papanicolaou
PSA: prostate-specific antigen
UIBE: University of International Business and Economics
